# Practice Models and Challenges in Teledermatology: A Study of Collective Experiences from Teledermatologists

**DOI:** 10.1371/journal.pone.0028687

**Published:** 2011-12-14

**Authors:** April W. Armstrong, Mei W. Kwong, Lynda Ledo, Thomas S. Nesbitt, Sandra L. Shewry

**Affiliations:** 1 Department of Dermatology, University of California Davis School of Medicine, Sacramento, California, United States of America; 2 Center for Connected Health Policy, Sacramento, California, United States of America; 3 Office of the Dean, University of California Davis School of Medicine, Sacramento, California, United States of America; The University of Queensland, Australia

## Abstract

**Background:**

Despite increasing practice of teledermatology in the U.S., teledermatology practice models and real-world challenges are rarely studied.

**Methods:**

The primary objective was to examine teledermatology practice models and shared challenges among teledermatologists in California, focusing on practice operations, reimbursement considerations, barriers to sustainability, and incentives. We conducted in-depth interviews with teledermatologists that practiced store-and-forward or live-interactive teledermatology from January 1, 2007 through March 30, 2011 in California.

**Results:**

Seventeen teledermatologists from academia, private practice, health maintenance organizations, and county settings participated in the study. Among them, 76% practiced store-and-forward only, 6% practiced live-interactive only, and 18% practiced both modalities. Only 29% received structured training in teledermatology. The average number of years practicing teledermatology was 4.29 years (SD±2.81). Approximately 47% of teledermatologists served at least one Federally Qualified Health Center. Over 75% of patients seen via teledermatology were at or below 200% federal poverty level and usually lived in rural regions without dermatologist access. Practice challenges were identified in the following areas. Teledermatologists faced delays in reimbursements and non-reimbursement of teledermatology services. The primary reason for operational inefficiency was poor image quality and/or inadequate history. Costly and inefficient software platforms and lack of communication with referring providers also presented barriers.

**Conclusion:**

Teledermatology enables underserved populations to access specialty care. Improvements in reimbursement mechanisms, efficient technology platforms, communication with referring providers, and teledermatology training are necessary to support sustainable practices.

## Introduction

Teledermatology is the practice of delivering dermatological care via communication technology [1]–[4]. The two primary forms of teledermatology practiced in the United States are live-interactive (LI) and Store-and-Forward (S&F) teledermatology [5]–[7], and a few programs employ a “hybrid” model, where images captured through digital cameras are used in combination with videoconferencing [8].

Despite increasing practice of teledermatology in the U.S., teledermatology practice models in the various settings are rarely studied. It is often difficult for dermatologists new to teledermatology to efficiently gather relevant information regarding best practice models. Furthermore, these new practitioners may not be aware of the potential challenges that could undermine a sustainable teledermatology practice. Thus, an investigation on best practice models in teledermatology and a candid discussion of challenges of practicing teledermatology will be valuable to dermatologists, primary care providers, and policy makers.

Among the states that reimburse for LI and S&F teledermatology, California ranks top for having the most practicing teledermatologists and the highest volume of teledermatology consultations [9]. However, despite of the collective experience of these teledermatologists, no study has systematically examined teledermatology practice models and shared challenges.

The primary aim of this study is to examine teledermatology practice models and challenges in California focusing on its role in serving the Medicaid population. Specifically, we examined teledermatology practice operations, reimbursement considerations, practice challenges, and incentives. This study allows for identification of practice models and in-depth discussion of practice challenges, which will benefit both practitioners new to teledermatology and those seeking to improve their existing programs.

## Methods

### Study Setting

This study was approved by the Institutional Review Board at UC Davis. Using a multi-pronged approach, we sought to identify all dermatologists practicing teledermatology in California. We contacted the ATA Teledermatology Special Interest Group and the Telemedicine Task Force at the American Academy of Dermatology (AAD) to identify practicing teledermatologists in California. In addition, we submitted a Public Records Act request to the Department of Health Care Services (DHCS) to obtain Medicaid records to identify dermatologists who have submitted claims for teledermatology services from January 1, 2007 through December 31, 2009. We also leveraged the existing network of teledermatologists to identify other practicing teledermatologists who might not have been captured with the above outreach efforts in California.

### Instrument Development and In-Depth Interviews

We conducted in-depth hour-long interviews with practicing teledermatologists in California between September 1^st^ 2010 through March 30^th^ 2011. The interview questions were developed by the authors in collaboration with the committee members from the ATA Teledermatology SIG and the MediCal-Policy section of the California DHCS. The interview questions were revised in four iterations to ensure internal, external, and face validity. These interview questions focused on the following five areas: (A) demographic characteristics of teledermatologists and patients (B) operational considerations of the teledermatology practice, (C) reimbursement considerations, (D) practice challenges and areas of improvement, and (E) ways to incentivize other dermatologists to participate in teledermatology.

## Results

### A. Demographic Characteristics of Teledermatologists and Patients Cared through Teledermatology

A total of 14 dermatologists who practice teledermatology were initially identified. During the study, Kaiser Permanente in California launched their teledermatology programs. All directors of the Kaiser teledermatology programs agreed to participate in the study to yield a total of 17 teledermatologists. We conducted hour-long interviews with these 17 dermatologists who practiced teledermatology in California between 2007 and 2011. The average teledermatology experience in any state was 4.29 years (SD±2.81 years), and the average teledermatology experience in California was 3.85 years (SD±2.75 years).

**Figure 1:**
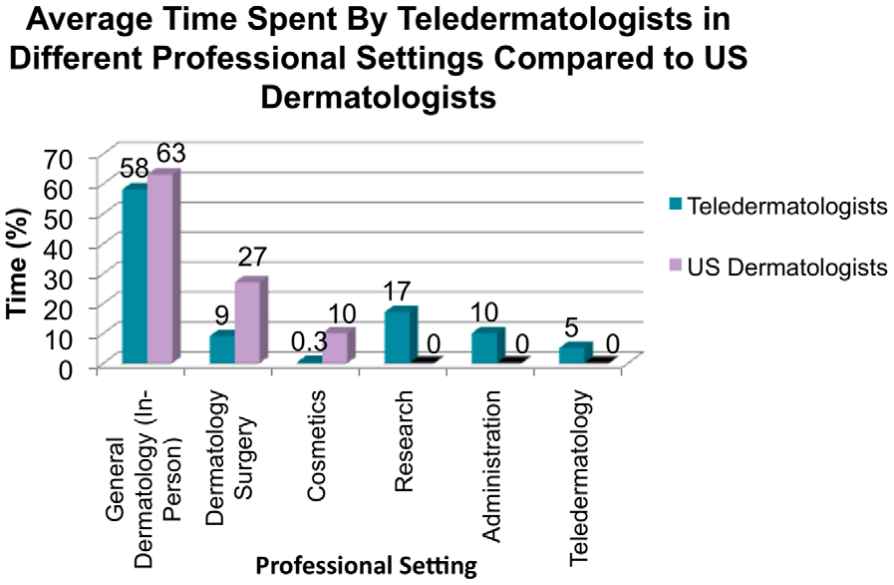
Comparison of Professional Effort by Teledermatologists and General U.S. Dermatology Workforce.

These teledermatologists spent a mean of 58% of their professional time in face-to-face medical dermatology, 9% time in surgery, 0.3% time in cosmetic dermatology, 17% time in research, 10% time in administration, and 5% time in teledermatology (Figure 1). Approximately 35% of the teledermatologists identified university-based setting as their primary practice setting; 24% identified private practice; 18% identified County-Hospitals; 18% identified managed care organizations; and 6% identified Veterans Administration Hospitals.

When asked if they received formal training in telehealth, including supervised practice or formal course training, 71% of teledermatologists reported that they did not receive training in telehealth, and 29% reported having received training. Among those who received teledermatology training, they obtained training through courses offered at ATA, teaching from experienced teledermatologists, and/or sessions with vendors of the software applications.

These dermatologists reported that their teledermatology patients comprised primarily of rural and indigent populations. Compared with the general population, no particular racial or ethic groups were over-represented in the teledermatology patient population. Rather, the populations served by teledermatology tended to be indigent and from rural geographic areas. More than 75% of patients cared for through teledermatology were those at or below 200% federal poverty level, and they usually lived in geographically isolated regions without ready access to dermatologists.

The teledermatologists were asked to report the average volume of Medicaid patients that they served in one month. The teledermatologists cared for a mean of 8.1 Medicaid patients (SD±4.4 patients) per month through teledermatology consultations. Approximately 47% of the teledermatologists served at least one Federally Qualified Health Center or safety-net clinic via teledermatology.

### B. Operational Considerations of Teledermatology Practice

#### Health Care Delivery Models

The teledermatologists reported that the most important advantages for practicing S&F teledermatology were increased efficiency (59%), increased access convenience (53%), increased patient satisfaction (53%), increased referring provider satisfaction (35%), timely care (35%), and cost-effective care (18%).

**Figure 2:**
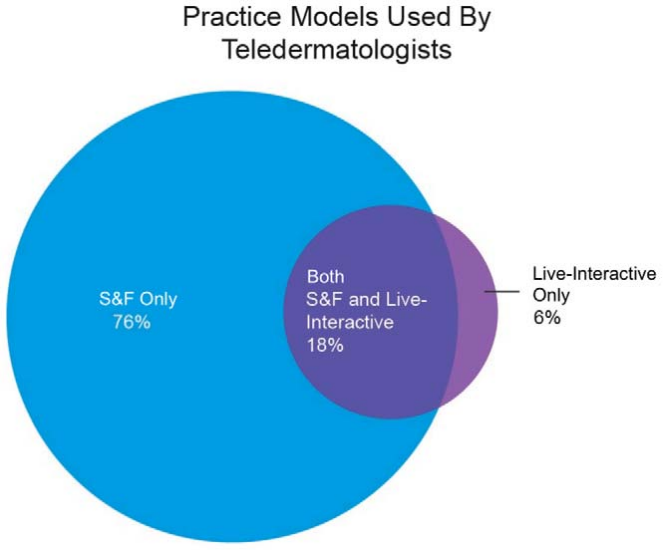
Technology-Based Teledermatology Practice Patterns among Teledermatologists in California.

Of the 17 teledermatologists, 76% practiced S&F teledermatology only, 6% practiced live-interactive teledermatology only, and 18% practiced a combination of S&F and live-interactive teledermatology (Figure 2). The teledermatologists spent a mean of 4.4 hours per week on completing a mean of 23 S&F or LI reimbursable consults. Thirty-five percent also provided *pro bono* volunteer teledermatology consultations, and 65% did not provide volunteer consultations regularly.

The teledermatologists were asked if their S&F teledermatology recommendations were used for triage, consultation, direct care of patients, or any combination of the above purposes [10]. In the triage model, teledermatologists review all new referrals for dermatology, prioritize and determine timing for patients requiring in-person consultations, and provide brief recommendations to the primary care providers. The consultative model is the most widely practiced teledermatology model to date. In the consultative model, the dermatologists serve as consultants and provide detailed recommendations after reviewing the clinical history and images. The dermatologists do not provide direct care for the patients; rather, the primary care providers decide whether to implement the dermatologists' recommendations and assume full care of the patients. In the direct-care model, the patients seek and receive treatments directly from the specialists. Because no specific reimbursement mandates exist in most U.S. states for the direct-care teledermatology model at the current time, this model incur out-of-pocket expenses in commercial settings and is being evaluated in research settings [11], [12]. Approximately 53% of teledermatologists perform S&F teledermatology for the sole purpose of providing consultations; 23% use S&F teledermatology for the sole purpose of triage; 23% reported dual purposes of both triage and consultations, and 12% reported combination of triage, consultations, and direct care of patients.

#### Value of Providing Teledermatology Care to MediCaid Population

The teledermatologists were asked to provide the perceived values of providing S&F teledermatology to serve the Medicaid population in California. The teledermatologist-reported that teledermatology services were associated with increased efficiency (59%), increased access (47%), increased patient satisfaction (35%), timely and quality patient care (18%), cost-effective care (12%), and enhanced referring provider satisfaction (6%).

When asked if they would like to provide more or less teledermatology services to their Medicaid population, all teledermatologists reported that they would like to provide more teledermatology services. All teledermatologists expressed that teledermatology is a valuable and efficient process for providing specialty care to the Medicaid population.

#### Staffing Requirements and Teledermatology Applications

With regards to expected staffing requirement on the referral sites, nearly 94% of the teledermatologists reported no specific staffing requirements for the referral sites. The referral sites used a variety of personnel with varying levels of medical training to staff the teledermatology clinic, which included medical assistants, physician assistants, nurses, administrative assistants, and information technologists. One teledermatologist indicated that licensed physicians were required to obtain the history and transmit the photographs.

The teledermatologists used a variety of software applications for completing and transmitting consultations, with Second Opinion being the most frequently used application (59%). The software applications used by the teledermatologists included Second Opinion, MedWeb, Telederm Solutions, AFHCAN, ClickDiagnostics, Direct Dermatology, and applications internal to respective health systems.

#### Follow-Up of Teledermatology Patients

In some instances, after a teledermatologist evaluates a clinical case, he or she may decide that the patient needs to see a dermatologist in-person. This is usually due to one of the following reasons. First, the images were inadequate for teledermatology evaluations. Second, the patient may benefit from a more thorough evaluation by a dermatologist in-person, such as a full-body skin check. Finally, the teledermatologist may recommend that the patient undergo a procedure with a dermatologist in-person.

When asked how these teledermatologists handled follow-up visits when in-person evaluation with a dermatologist is necessary, all teledermatologists responded that they recommended follow-up with a local dermatologist in-person. All teledermatologists did not require that the patient follow up with the consulting teledermatologist in-person, unless the referring physician could not locate a local dermatologist and would like the patient be followed up with the teledermatologist.

#### Practice Efficiency

One of the perceived primary advantages of S&F teledermatology has been increased efficiency compared to in-person evaluations. When asked whether S&F teledermatology was as efficient as in-person consultations for medium-complexity cases, 88% of the teledermatologists reported that S&F teledermatology was more efficient than in-person evaluations, and 12% reported that it was less efficient. For S&F teledermatology consults of medium complexity, the teledermatologists spent a mean of 9.4 minutes to complete the S&F consultation (SD±5.2 minutes).

#### Skin Diseases Less Suited for Teledermatology

The majority (59%) of teledermatologists reported that all skin diseases were amenable to S&F and live-interactive teledermatology, and 41% reported that some conditions were not suitable for teledermatology. Conditions reported to be not suitable for teledermatology included full-body skin examinations, lesions in the hair-bearing area, melanocytic lesions in high-risk patients, and patients with diagnosis of melanoma that required in-person counseling.

### C. Reimbursement Considerations and Building a Sustainable Practice

In general, this group of teledermatologists lacked specific knowledge of the financial operations and reimbursement landscape of the teledermatology operation. Among the 81% of teledermatologists who reported performing consultations on a contractual basis, the average number of clinics served per teledermatologist was 2.2 (SD±5.2) clinics. Among these clinics, at least 1.7 (SD±2.1) clinics had uninsured patients.

When asked about the success rate in obtaining reimbursement for their teledermatology services from Medicaid, 53% of teledermatologists reported a mean success rate of 41% (SD±29%). When asked how reimbursement success for teledermatology differed from in-person evaluations for the Medicaid population, 35% of the teledermatologists reported that reimbursement for teledermatology was lower than that of in-person care per clinic, and 65% reported that they did not have sufficient information for that comparison. No teledermatologist reported that teledermatology was reimbursed comparable or better than in-person encounters per clinic.

The teledermatologists identified one or more of the following factors as important for making their teledermatology practice sustainable: streamlined practice model with consistent staff and efficient software application (88%), a sustainable business model (47%), collegial relationship between dermatologists and referring providers (47%), adequate training of the referral sites (41%), and high-resolution images (12%).

### D. Practice Challenges and Areas of Improvement

The teledermatologists reported one or more of the following factors as being challenging in their teledermatology practices: obtaining reimbursement (71%), resolving technology-related issues (65%), communicating with referring providers effectively (41%), setting-up teledermatology operation and training teledermatology staff (41%), and following up with medically complex patients (12%).

**Figure 3:**
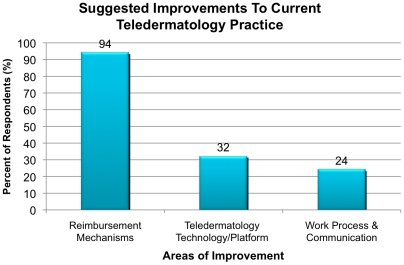
Priority Areas of Improvements in Teledermatology.

In order to make meaningful strides towards improving special access to patients, we need to make specific recommendations based on identified challenges. Approximately 94% of teledermatologists recommended improvements in reimbursement mechanisms (Figure 3). Specific recommendations included increasing awareness among insurers of “reimbursability” of teledermatology and timely reimbursement of teledermatology services. A total of 32% reported that they would like to see improvements in the technologies used for S&F and LI teledermatology. Finally, 24% of the respondents reported that streamlined work processes and improved communication with PCPs are necessary. Specifically, some teledermatologists who performed S&F teledermatology expressed that they did not know the extent to which their recommendations were relayed to the patients by the PCPs.

### E. How to Incentivize Other Dermatologists to Participate in Teledermatology

Even though various forms of teledermatology have existed since the 1970s, this healthcare delivery method has not yet experienced widespread adoption. We also asked the teledermatologists regarding effective means of incentivizing other dermatologists to provide care to Medicaid population via telehealth.

Approximately 94% of teledermatologists stated that financial incentives were the key to encouraging other dermatologists to participate in telehealth for Medicaid population. Specifically, a speedy and uncomplicated reimbursement process for teledermatology and federal loan repayment programs for those providers engaged in teledermatology for underserved populations were important. Approximately 88% also cited improved efficiency in workflow, including an easy-to-use technology optimized for physician convenience, was necessary to incentivize other dermatologists. Nearly 53% of the respondents stated the removal of legal liability for teledermatology consultations as a means of incentivize provision of care to uninsured populations. Finally, 53% of the respondents reported increasing awareness among dermatologists and educating dermatology residents in teledermatology were important.

## Discussion

Teledermatology has been reported to improve patient access, provide cost-effective care, and increase distance medical education [1], [3], [13]–[19]. However, a gap exists between the reported benefits of teledermatology and its relatively slow adoption in clinical practice. Part of the challenges for newcomers to teledermatology is the lack of literature on practice models and shared challenges associated with teledermatology practices.

Legislations regulating provision of telemedicine services are set by individual states. In California, credentialing criteria are set by the Joint Commission, CMS, and Title 22 from the California Department of Public Health. In California, the Telemedicine Development Act of 1996 (SB 1665) defines the major requirements and payment for telemedicine services [20]. A key provision in this act requires all insurance carriers to reimburse LI Telemedicine services. CMS sets its own regulations for reimbursement of telemedicine services. Whereas both the Medicare and Medi-caid program in California provide reimbursement for LI teledermatology, Medicare does not at this time provide reimbursement to S&F teledermatology. CMS generally requires the use of telemedicine modifier “GT” for live-interactive telemedicine and “GQ” for S&F telemedicine.

The passage of California's assembly bill AB 415 is expected to increase access to telemedicine starting January 1, 2012 through the following means [21]. Key provisions of the bill include (1) updated definition of telehealth to reflect the broader range of services in use today and application of the definition to all licensed health professionals, (2) replacement of written consent with verbal consent, (3) removal of the Medi-Caid rule requiring documentation of a barrier to an in-person visit before a beneficiary can receive telehealth services, (4) inclusion of S&F technologies as viable for all types of telehealth services, and (5) elimination of restrictions on reimbursement of services provided via email or telephone.

According to the 2010 American Medical Association report “Physician Characteristics and Distribution in the US”, a total of 1,594 board-certified and licensed dermatologists practice in the state of California [22]. This is the first study to date that examined the collective experience of teledermatologists in California with an emphasis on practice models and challenges. This study may serve as a catalyst to identify opportunities to increase teledermatology adoption in the dermatology community at large.

### A. Comparison of Teledermatologists with the Overall U.S. Dermatology Workforce

Do teledermatologists differ in how they spend their time professionally from the general U.S. dermatology workforce? In 2007, an average dermatologist in the U.S. spends 63% of time in medical dermatology, 27% time in surgery, and 10% in cosmetic dermatology [23]. While the teledermatologists in California spent a similar amount of time in face-to-face medical dermatology, they spent significantly less time in surgical and cosmetic dermatology and more time in research and administration (Figure 1). These differences in efforts may reflect varying subspecialty interests and priorities in serving different patient populations.

### B. Teledermatology Practice Models and Operational Considerations

Most teledermatologists who participated in this study performed S&F teledermatology either exclusively or in combination with LI teledermatology. The preponderance of S&F modality could be owing to the lower equipment cost, lower administrative overhead, and potentially higher efficiency compared to LI modality.

For teledermatologists engaged in S&F practice, improving practice efficiency was paramount to a sustainable operation. Because the referral sites invest time to capture skin images and obtain history, choosing the right staff to serve as teledermatology coordinators is important. While some teledermatologists may prefer teledermatology coordinators to have substantial medical background, the results of this study indicate that most teledermatologists do not have specific requirements. In practice, medical assistants, physician assistants, nurses, administrative assistants, and information technologists have all been employed as teledermatology coordinators at various sites. This may reflect the lack of regular staffing available at the referral sites to image patients. Therefore, it is important that all teledermatology coordinators receive adequate training on imaging and taking relevant medical history.

While most teledermatologists perceived S&F teledermatology to be more efficient than in-person consultations, a minority thought that S&F was less efficient than in-person clinic. The primary cited reason for decreased efficiency was poor image quality and/or inadequate clinical history. As a result, the teledermatologist was unable to make a diagnosis based on the available information, and additional time was spent communicating with the referral site to obtain additional history or request re-imaging. In addition, due to the asynchronous nature of the encounter, some teledermatologists reported that follow-up questions from either referring provider or the patient obviated the apparent efficiency of the initial S&F evaluations. Therefore, training of a dedicated teledermatology coordinator and clear, specific recommendations from the teledermatologists are important for practice efficiency.

### C. Practice Barriers and Suggestions for Addressing Challenges

The challenges of practicing teledermatology are not extensively explored in the current literature [24]–[26]. An in-depth understanding of the challenges of practicing teledermatology enables the policy makers, specialists, and referring providers to make purposeful improvements in the system.

#### Reimbursement for Teledermatology Services

As this study indicated, a perception exists that consultations delivered through either S&F or LI teledermatology are reimbursed at a lower rate compared to in-person services. However, this is not the case in California when we compared specific Medicaid and Medicare rates for telehealth services with that from in-person encounters. That is, to date, for the *same* level of consultative service in California, S&F or LI teledermatology reimbursements are reimbursed at the same rate as that for in-person encounters for Medicaid and Medicare. A possible explanation for the perceived lower reimbursement associated with teledermatology might be attributed to the lack of procedures (such as cryotherapy or biopsies) during the teledermatology encounter. The lack of procedures likely resulted in lower reimbursement in teledermatology per encounter compared to in-person visits where procedures occur.

Among the challenges reported by teledermatologists, obtaining reimbursement for teledermatology services ranked top. These difficulties ranged from delays in reimbursements to not being reimbursed at all for the teledermatology consultations. Some teledermatologists reported that some insurers did not recognize telemedicine-specific modifiers (such as GT or GQ) associated with the claims, which resulted in delay and sometimes non-payment.

Nearly all teledermatologists urged that improvements in the reimbursement mechanisms are necessary. Possible ways of addressing these reimbursement issues include educating insurers of “reimbursability” of teledermatology and advocate for timely reimbursement of teledermatology services. However, education efforts can be time-consuming and potentially sporadic. Alternatively, eliminating modifiers for telemedicine services will likely result in timely and more uniform improvement in reimbursement for telemedicine services.

#### Overcoming Technological Barriers

Technological challenges were the second most cited challenge among teledermatologists. These challenges included inefficient and expensive software programs, platforms that do not integrate with existing electronic medical record systems, and poor image quality. The commercially available S&F telemedicine platforms are often too expensive for the referral sites and/or teledermatologists to purchase [27], [28]. Thus, making S&F applications affordable and able to integrate with existing electronic medical record systems will be helpful. In addition, although new consumer-grade digital cameras are often adequate for capturing digital images, without adequate training of the teledermatology staff, the teledermatologists could receive images of poor quality, which significantly impairs their ability to provide high-quality and timely care. Therefore, standardized training and continued training of teledermatology coordinators, especially in imaging, is essential to providing quality images.

Innovations in technology are necessary to provide streamlined and efficient telemedicine care. Advances in diagnostic decision support systems and mobile technology are ushering a new wave of meaningful technology purported to improve patient care. For example, a visual diagnostic decision support system (such as VisualDx) can be used to support both primary care physicians and specialists in telemedicine [29]. The specialist can extract information from this decision support system to provide up-to-date recommendations more efficiently and educate the referring providers on possible differential diagnoses related to the current patient.

#### Communication with Referring Providers

The asynchronous nature of S&F teledermatology is useful for practice efficiency and reducing overhead; however, inherent challenges with asynchronous communication may present challenges for the referring physicians and dermatologists. Often times, because the sole form of communication is the dermatologist's written recommendations, the asynchronous format does not lend itself readily to exchanges among the providers or with the patient. For example, the teledermatologists may not know the extent that their recommendations are actually implemented or communicated to the patients. This lack of feedback and exchange could prevent teledermatology programs from growth and improvement. Therefore, the teledermatologists should encourage PCPs and patients seek clarification if they have questions regarding the recommendations. This type of exchange will not only result in improved patient care; it will also allow PCPs to learn from difficult-to-manage dermatology diseases.

Defining incentives for referring provider participation in telemedicine is important for sustainability of the program. Telemedicine enables referring providers to increase patient access to specialists, retain patients in their own communities for follow-up care, and obtain patient-based medical education.

### D. Future Directions

As communications technology continues to connect medical expertise with patients, continued adoption of teledermatology needs to be grounded in quality and efficient patient-care processes. Documentation of the various teledermatology practice models and shared challenges will help propel the field forward by identifying areas of improvement.

In the coming years, overcoming the challenges of poor-quality images, delayed reimbursement, and potentially higher medico-legal risks will be important to address. Furthermore, technological advancements in creating affordable and versatile platforms for teledermatology will be necessary to improve practice efficiency. Finally, educating trainees on practicing synchronous and asynchronous teledermatology will help with continued efforts of increasing dermatology access to patients in geographically remote and medically underserved communities.

## References

[1] Edison KE, Ward DS, Dyer JA, Lane W, Chance L (2008). Diagnosis, diagnostic confidence, and management concordance in live-interactive and store-and-forward teledermatology compared to in-person examination.. Telemedicine journal and e-health : the official journal of the American Telemedicine Association.

[2] Krupinski E, Burdick A, Pak H, Bocachica J, Earles L (2008). American Telemedicine Association's Practice Guidelines for Teledermatology.. Telemedicine journal and e-health : the official journal of the American Telemedicine Association.

[3] Johnson MN, Armstrong AW (2011). Technologies in dermatology: teledermatology review.. Giornale italiano di dermatologia e venereologia : organo ufficiale, Societa italiana di dermatologia e sifilografia.

[4] Pathipati AS, Lee L, Armstrong AW (2011). Health-care delivery methods in teledermatology: consultative, triage and direct-care models.. Journal of telemedicine and telecare.

[5] Warshaw EM, Hillman YJ, Greer NL, Hagel EM, Macdonald R (2010). Teledermatology for diagnosis and management of skin conditions: A systematic review.. Journal of the American Academy of Dermatology.

[6] Warshaw E, Greer N, Hillman Y, Hagel E, MacDonald R (2010). Teledermatology for Diagnosis and Management of Skin Conditions: A Systematic Review of the Evidence..

[7] Pak H, Triplett CA, Lindquist JH, Grambow SC, Whited JD (2007). Store-and-forward teledermatology results in similar clinical outcomes to conventional clinic-based care.. J Telemed Telecare.

[8] Pak H, Edison K, Whited J Teledermatology: A User's Guide:.

[9] Group ATATSI (2003). ATA U.S. Teledermatology Program Survey 2003.. American Telemedicine Association.

[10] Pathipati AS, Lee L, Armstrong AW (2011). Health-care delivery methods in teledermatology: consultative, triage and direct-care models.. J Telemed Telecare.

[11] Chambers CJ, Parsi KK, Schupp C, Armstrong AW (2011). Patient-centered online management of psoriasis: A randomized controlled equivalency trial.. Journal of the American Academy of Dermatology.

[12] Parsi KK CC, Armstrong AW (2011).

[13] Whited JD (2006). Teledermatology research review.. Int J Dermatol.

[14] Eminovic N, de Keizer NF, Bindels PJ, Hasman A (2007). Maturity of teledermatology evaluation research: a systematic literature review.. Br J Dermatol.

[15] Quinley KE, Gormley RH, Ratcliffe SJ, Shih T, Szep Z (2011). Use of mobile telemedicine for cervical cancer screening.. Journal of telemedicine and telecare.

[16] Tran K, Ayad M, Weinberg J, Cherng A, Chowdhury M (2011). Mobile teledermatology in the developing world: implications of a feasibility study on 30 Egyptian patients with common skin diseases.. Journal of the American Academy of Dermatology.

[17] Colven R, Shim MH, Brock D, Todd G (2011). Dermatological diagnostic acumen improves with use of a simple telemedicine system for underserved areas of South Africa.. Telemedicine journal and e-health : the official journal of the American Telemedicine Association.

[18] Armstrong AW, Dorer DJ, Lugn NE, Kvedar JC (2007). Economic evaluation of interactive teledermatology compared with conventional care.. Telemedicine journal and e-health : the official journal of the American Telemedicine Association.

[19] Chen TS, Goldyne ME, Mathes EF, Frieden IJ, Gilliam AE (2010). Pediatric teledermatology: observations based on 429 consults.. Journal of the American Academy of Dermatology.

[20] (1996). Bill Number: SB 1665 Chaptered.. http://leginfo.ca.gov/pub/95-96/bill/sen/sb_1651-1700/sb_1665_bill_960925_chaptered.html.

[21] (2011). Bill Number: AB 415 Chaptered.. http://leginfo.ca.gov/pub/11-12/bill/asm/ab_0401-0450/ab_415_bill_20111007_chaptered.html.

[22] Smart DR (2010).

[23] Kimball AB, Resneck JS (2008). The US dermatology workforce: a specialty remains in shortage.. J Am Acad Dermatol.

[24] Warshaw EM, Gravely AA, Nelson DB (2010). Accuracy of teledermatology/teledermoscopy and clinic-based dermatology for specific categories of skin neoplasms.. Journal of the American Academy of Dermatology.

[25] Warshaw EM, Gravely AA, Bohjanen KA, Chen K, Lee PK (2010). Interobserver accuracy of store and forward teledermatology for skin neoplasms.. Journal of the American Academy of Dermatology.

[26] Warshaw EM, Lederle FA, Grill JP, Gravely AA, Bangerter AK (2009). Accuracy of teledermatology for nonpigmented neoplasms.. Journal of the American Academy of Dermatology.

[27] Armstrong AW, Sanders C, Farbstein AD, Wu GZ, Lin SW (2010). Evaluation and comparison of store-and-forward teledermatology applications.. Telemedicine journal and e-health : the official journal of the American Telemedicine Association.

[28] Edison KE (2007). The future of dermatology: challenges and opportunities.. Missouri medicine.

[29] David CV, Chira S, Eells SJ, Ladrigan M, Papier A (2011). Diagnostic accuracy in patients admitted to hospitals with cellulitis.. Dermatology online journal.

